# MicroRNA‐204‐5p: A pivotal tumor suppressor

**DOI:** 10.1002/cam4.5077

**Published:** 2022-07-31

**Authors:** Fan Yang, Zehua Bian, Peiwen Xu, Shengbai Sun, Zhaohui Huang

**Affiliations:** ^1^ Wuxi Cancer Institute Affiliated Hospital of Jiangnan University Wuxi Jiangsu China; ^2^ Laboratory of Cancer Epigenetics, Wuxi School of Medicine Jiangnan University Wuxi Jiangsu China

**Keywords:** microRNA, miR‐204‐5p, tumor suppressor, cancer, gene regulation

## Abstract

MicroRNAs (miRNAs) are a class of non‐coding single‐stranded RNA molecules with a length of approximately 18‐25 nt nucleotides that regulate gene expression post‐transcriptionally. MiR‐204‐5p originates from the sixth intron of the transient receptor potential cation channel subfamily M member 3 (TRPM3) gene. MiR‐204‐5p is frequently downregulated in various cancer types and is related to the clinicopathological characteristics and prognosis of cancer patients. So far, many studies have determined that miR‐204‐5p functions as a tumor suppressor for its extensive and powerful capacity to inhibit tumor proliferation, metastasis, autophagy, and chemoresistance in multiple cancer types. MiR‐204‐5p appears to be a promising prognostic biomarker and a therapeutic target for human cancers. This review summarized the latest advances on the role of miR‐204‐5p in human cancers.

## Graphical abstract

MiR‐204‐5p regulates tumorigenesis and progression via various target genes in human cancers.

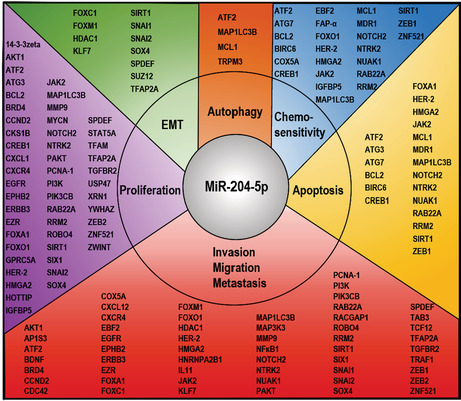

## 1 INTRODUCTION

The occurrence and development of cancer are dynamic and complex and involve a variety of genetic and epigenetic mechanisms. MicroRNAs (miRNAs) are a class of small endogenous regulatory RNAs (18‐25 nt) lacking protein‐coding abilities. MiRNAs have been widely identified in multiple species and play an important role in the development and diseases.^1^ By binding to the 3'untranslated regions (3'UTRs) of their mRNA targets, miRNAs participate in the formation of RNA‐induced silencing complex and inhibit the expression of target mRNAs. Now, it is clear that the base‐pairing between miRNAs and their mRNA targets could induce mRNA degradation or translational repression.^2^ In addition to the classical mechanism by which miRNAs inhibit mRNA expression, some non‐coding RNAs (ncRNAs), such as long non‐coding RNAs (lncRNAs) and circular RNAs (circRNAs), also could be bound and regulated by miRNAs. MiRNAs and their RNA targets form a regulatory network named the competitive endogenous RNA (ceRNA) network.^3^

MiR‐204‐5p, previously named miR‐204 in the miRBase database before the release 19.0 (https://www.mirbase.org/),^4^ originates from TRPM3 intron 6. Since miR‐204‐5p was discovered, numerous studies have revealed its key role in many essential physiological or pathological processes, especially tumorigenesis and progression. In normal tissues, miR‐204‐5p has been demonstrated to play a significant role in eye development,^5^, ^6^ lipogenesis,^7^ and osteogenesis.^8^ For instance, overexpression of miR‐204‐5p in the human retinal epithelium governs eye development. MiR‐204‐5p has also been shown to stimulate the development of human adipose mesenchymal stem cells into mature adipocytes.^9^ Besides, by modulating the IL6/IL6R axis, miR‐204‐5p reduces inflammation, and chemokine production in renal tubular epithelial cells.^10^

Increasing pieces of evidence have expounded that miR‐204‐5p functions as a key tumor suppressor in a variety of human tumors by regulating proliferation, stemness, metastasis, apoptosis, chemoresistance, and autophagy. Additionally, clinical analyses reveal that miR‐204‐5p expression is in relation to the prognosis and clinicopathological characteristics of cancer patients. In this review, we summarized the latest advances regarding the pivotal suppressive role of miR‐204‐5p in various tumor types and discussed its potential applications in cancer therapy.

## 2 ABNORMAL EXPRESSION OF MIR‐204‐5P IN HUMAN CANCERS

Generally, miR‐204‐5p plays a tumor inhibitory role and decreased expression of miR‐204‐5p promotes tumorigenesis and progression. Although a limited number of studies reported that miR‐204‐5p was overexpressed in prostate cancer (PCa),^11^ breast cancer (BC),^12^ and ovarian cancer (OC),^13^ most studies showed that the expression of miR‐204‐5p is significantly decreased in a series of cancer types, including the abovementioned PCa,^14^ BC,^15^, ^16^, ^17^and OC.^18^

The expression of miR‐204‐5p could be regulated at transcriptional and post‐transcriptional levels. Firstly, DNA methylation has been shown to epigenetically silence miR‐204 in human cancers.^19^, ^20^, ^21^, ^22^ The miR‐204 coding sequence, located in the intron 6 of TRPM3, is produced from the same transcription unit as TRPM3 and has the same transcriptional regulatory motif.^23^, ^24^ It has been reported that the promoter of TRPM3/miR‐204 is hypermethylated in gliomas^21^ and colorectal cancer (CRC),^22^ leading to silenced miR‐204 expression. Secondly, many reports have shown that some ncRNAs, including lncRNAs and circRNAs, can bind and suppress the expression and/or activity of miR‐204‐5p in oncocytes.^25^, ^26^, ^27^, ^28^, ^29^, ^30^ Besides, some transcriptional factors (i.e., STAT3 and TFAP2A) have also been proved to regulate miR‐204 expression in tumors.^31^, ^32^, ^33^, ^34^, ^35^

## 3 THE ROLE OF MIR‐204‐5P IN HUMAN CANCERS

### 3.1 Breast cancer

BC is the most commonly diagnosed cancer worldwide, surpassing lung cancer (LC) with a calculated 2.3 million new cases.^36^ A growing number of studies showed that miR‐204‐5p is decreased in BC and exerts tumor‐suppressive functions.^15^, ^16^, ^17^, ^25^, ^37^, ^38^, ^39^, ^40^ These studies have identified many miR‐204‐5p targets by which miR‐204‐5p inhibits BC development and progression. For example, SOX4, a BC stem cell‐specific marker that promotes EMT, tumor growth, and metastasis, has been established as a key target of miR‐204 in BC cells.^41^ In addition, miR‐204‐5p impairs the proliferation of BC cells via targeting TGFBR2, AKT, and PI3K.^37^ MiR‐204‐5p can also inhibit the progression of BC by inhibiting ANGPT1,^38^ AP1S3,^42^ BDNF,^43^ COX5A,^40^ IL‐11,^44^ PIK3CB,^16^ PTEN,^37^ and RRM2.^25^ Interestingly, Findlay et al. declared that miR‐204‐5p is upgraded in BC and facilitates tumor cell invasion and migration by targeting PDEF.^12^ However, in view of the very low case number of this study (*n* = 5), its conclusion of miR‐204‐5p downregulation in BC is subject to deliberation.

Recent studies showed that many lncRNAs are aberrantly expressed and play significant parts in human cancers. Some lncRNAs indirectly regulate gene expression through working as ceRNAs of miRNAs. For example, in triple‐negative breast cancer, lncRNA ARNILA acts as a ceRNA for miR‐204‐5p to promote the expression of its target genes, including SOX4, BCL2, RAB22A, SIRT1, and FOXA1.^39^ Interestingly, although RUNX2 was also a verified target of miR‐204‐5p, ARNILA knockdown, or overexpression had no effect on its expression in triple‐negative breast cancer,^39^ suggesting that the action of ARNILA on miR‐204‐5p is insufficient to concurrently inhibit all of its target genes. In addition, the expression of RUNX2 may be controlled by other regulatory mechanisms that counterbalance the impact of ARNILA on miR‐204.^39^ Wang et al. revealed that MALAT1 induces EMT phenotype and promotes metastasis via regulating the miR‐204/ZEB2 axis in BC.^45^ It has also been demonstrated that DSCAM‐AS1^25^ could bind miR‐204‐5p and upregulate its target RRM2, thus promoting BC growth and metastasis. Furthermore, DGUOK‐AS1 also acts as a ceRNA of miR‐204‐5p and promotes BC progression and metastasis.^46^

CircRNAs are endogenous ncRNAs with a covalently closed loop. Several circRNAs have been reported to regulate the targets of miR‐204‐5p via acting as a miR‐204‐5p sponge. For example, circPVT1 was reported to promote BC growth, EMT, and invasion via inhibiting miR‐204‐5p.^29^

### 3.2 Colorectal cancer

CRC is the third most commonly diagnosed cancer and the second leading cause of cancer death.^36^ We revealed that miR‐204‐5p is significantly downregulated in CRC, which was mediated by DNA hypermethylation of its promoter.^22^ We previously reported that RAB22A expression is elevated in CRC and represents an independent survival risk factor. What is more, we revealed, for the first time, that RAB22A is a key functional target of miR‐204‐5p and mediates its tumor‐inhibitory functions in CRC.^22^ We also elucidated that miR‐204‐5p inhibits CRC growth, metastasis, and chemoresistance by targeting CREB1.^28^ In addition, we revealed that tumor‐associated macrophages‐secreted IL‐6 induces chemoresistance through regulating the IL‐6R/STAT3/miR‐204‐5p axis in CRC cells.^34^ The tumor‐inhibitory effects of miR‐204‐5p in CRC have also been reported by other groups.^26^, ^47^

The methylation levels of TRPM3/miR‐204 promoter in CRC tissues were much higher than in corresponding non‐cancerous tissues, and the treatment with 5‐aza‐dC (a DNA methyltransferase inhibitor) restores miR‐204‐5p expression in CRC cells,^22^ suggesting DNA methylation is a key way to regulate the expression and function of miR‐204‐5p in CRC. LncRNAs also regulate miR‐204‐5p in CRC. We demonstrated, for the first time, that lncRNA—UCA1 accelerates proliferation and induces 5‐fluorouracil (5‐FU) resistance in CRC through binding to miR‐204‐5p and then increasing the expression of its targets (CREB1, BCL2, and RAB22A).^28^ Besides, Lu et al. showed that DSCAM‐AS1 promotes cell proliferation and migration by regulating the miR‐204‐5p/SOX4 axis in CRC.^47^ Jia et al. demonstrated that PlncRNA‐1 promotes CRC cell proliferation as well as liver metastasis by regulating the miR‐204/Wnt/β‐catenin axis.^48^ The abnormal overexpression of PCAT6 restrains miR‐204 expression, thus enhancing the activity of the HMGA2/PI3K axis, and finally induces the chemotherapeutic resistance of CRC cells to 5‐ FU.^26^

### 3.3 Prostate cancer

PCa ranks the second most common cancer in men.^36^ The main threat to PCa patients is the high rate of bone metastasis. PCa patients with bone metastasis showed significantly higher levels of miR‐204‐5p in tumor tissues and serum than those without bone metastasis.^14^ Functionally, several studies have confirmed that miR‐204‐5p suppresses PCa growth and metastasis. For example, Wa et al. uncovered that miR‐204‐5p inhibits PCa bone metastasis by targeting multiple targets (MAP3K3, TAB3, and TRAF1) and then inactivating NF‐κB signaling.^14^ In addition, miR‐204‐5p was also verified to promote apoptosis and chemosensitivity in PCa cells by downregulating BCL2^49^ and SIRT1.^50^ Moreover, Ding et al. demonstrated that the androgen receptor (AR)/miR‐204/XRN1 axis has dual regulatory effects on the growth of different PCa cells.^51^ Interestingly, Turner et al. confirmed the overexpression of miR‐204‐5p in PCa tissues^11^ and declared that miR‐204‐5p promotes PCa cell proliferation via suppressing PDEF. However, in this study, the case number is too small (*n* = 5) to prove the upregulation of miR‐204‐5p in PCa.

Chemoresistance is a key factor leading to tumor relapse and poor prognosis in human cancers, including PCa. We previously revealed that lncRNA—UCA1 promotes CRC tumorigenesis and chemoresistance by binding to miR‐204‐5p and restoring the expression of its target genes.^28^ Similarly, Wang et al. reported that UCA1 modulates the sensitivity of PCa cells to docetaxel by regulating the miR‐204/Sirt1 axis.^52^ Other groups also showed that UCA1 promotes tumor progression by acting as a ceRNA of miR‐204 and increasing the levels of ATF2 and CXCR4 in PCa.^53^, ^54^ In addition, lncRNA‐NEAT1 was found to promote docetaxel resistance by sponging miR‐204‐5p and then increasing ACSL4 expression in PCa.^55^

### 3.4 Gastric cancer (GC)

GC is the fifth most commonly diagnosed cancer.^36^ Many researchers have shown that miR‐204‐5p inhibits tumorigenesis and progression through regulating multiple targets in GC,^56^, ^57^, ^58^, ^59^, ^60^ including CKS1B, CXCL1, CXCL12, CXCR4, ERBB3, GPRC5A, RAB22A, USP47, and ZNF52. For example, Our group showed that miR‐204‐5p suppresses GC growth by targeting RAB22A and USP47.^58^ Extensive invasion and lymphatic metastasis is a key feature of advanced GC. Zhang et al. observed, for the first time, that the levels of miR‐204‐5p significantly decreased in tumor tissue and serum samples of GC patients, especially in those with lymphatic metastasis.^60^ Further functional and mechanistic investigations revealed that miR‐204‐5p inhibits GC metastasis via impairing CXCL12 and CXCR4.^60^ Besides, SOX4^61^ and SIRT1^62^ have also been reported as miR‐204‐5p key targets in regulating EMT, anoikis resistance and metastasis in GC.

Some lncRNAs, including BCYRN1,^63^ DLX6‐AS1,^64^ SNHG4,^65^ and LINC01234^66^ have been reported to be overexpressed in GC and inhibit the anti‐tumor function of miR‐204‐5p. For example, LINC01234 sponges miR‐204‐5p to upregulate CBFB expression, promoting GC tumorigenesis.^66^ In addition, Liang et al. uncovered a positive regulatory loop of DLX6‐AS1/miR‐204‐5p/OCT1 that promotes GC progression.^64^ CircRNAs also regulate miR‐204‐5p activity in GC. For instance, circSLAMF6 binds miR‐204‐5p and regulates the miR‐204‐5p/MYH9 axis in GC, thus facilitating cell migration invasion and glycolysis.^67^

### 3.5 Lung cancer

LC remains the leading cause of cancer death worldwide.^36^ JAK2/STAT3 pathway is a common signaling pathway with important regulatory functions in cell proliferation, differentiation, hemopoiesis, inflammation, and embryonic development. Aberrantly increased JAK2/STAT3 activity is frequently observed in a series of cancer types, including LC. Wang et al. demonstrated that miR‐204 suppresses cell migratory and invasive capacities in non‐small cell lung cancer (NSCLC) by inhibiting JAK2.^68^ Later, Liu et al. elucidated that miR‐204 inhibits angiogenesis in LC by regulating the JAK2‐STAT3 pathway.^69^ Interestingly, we previously revealed that STAT3 could transcriptionally repress miR‐204‐5p in CRC, indicating the involvement of a negative feedback loop.^34^ In addition, miR‐204‐5p inhibits cell proliferation, migration, and invasion in NSCLC by downregulating NUAK1^70^ and SIX1.^71^ Lastly, lncRNA—NEAT1 acts as a ceRNA of miR‐204‐5p to enhance the expression of NUAK1, resulting in increased cell proliferation, migration, and invasion in NSCLC.^72^ Similarly, lncRNA—MALAT1 functions as a sponge of miR‐204‐5p to enhance the expression of SLUG in lung adenocarcinoma, promoting EMT and metastasis.^73^

### 3.6 Liver cancer

Liver cancer is the sixth most common cancer, and hepatocellular carcinoma (HCC) accounts for more than 90% of primary liver cancer.^36^ MiR‐204‐5p also plays a critical role in regulating the development and progression of liver cancer. In HCC, multiple genes, including BCL2,^74^ NUAK1,^75^ SIR,T1^76^ and SIX1,^77^ have been identified as miR‐204‐5p targets, through which miR‐204‐5p exerts tumor‐suppressive functions.

Interestingly, miR‐204‐5p inhibits HCC cell proliferation by inhibiting HOTTIP, an oncogenic lncRNA.^78^ In addition, NEAT1 also counteracts the tumor‐inhibitory activity of miR‐204 by acting as a miR‐204 sponge in HCC.^79^ SNHG6 promotes cell cycle transition and tumorigenesis in HCC by suppressing miR‐204‐5p‐mediated inhibition of E2F1.^80^

In intrahepatic cholangiocarcinoma, miR‐204‐5p facilitates chemotherapeutic drug‐triggered apoptosis and inhibits proliferation via downregulating BCL2^81^ and SLUG.^82^ It has been shown that MALAT1 interacts with miR‐204‐5p to increase CXCR4 expression, leading to enhanced cell proliferation and invasion in hilar cholangiocarcinoma.^83^ Tu et al. reported that circ_0021205 sponges miR‐204‐5p and promotes RAB22A expression, thus promoting tumorigenesis in cholangiocarcinoma.^30^

### 3.7 Glioma

Gliomas are the most common brain tumors. Of them, glioblastoma is a diffuse, highly invasive tumor with poor clinical outcomes. Due to the promoter hypermethylation, miR‐204 was significantly downregulated in glioma.^21^, ^84^ Ying et al. showed that restoring the expression of miR‐204 simultaneously suppressed stem cell‐like phenotypes and migration of glioma cells by targeting SOX4 and EphB2.^21^ In addition, miR‐204 suppresses the development and progression of glioma by targeting ATF2,^85^ BCL2,^86^ ezrin,^87^ FAP‐α,^88^ CYP27A1,^89^ RAB22A^84^ or ZEB1.^90^ Several lncRNAs, including XIST,^86^ UCA1,^90^ and HOXD‐AS1,^91^ were also reported to bind and inhibit miR‐204‐5p in glioma.

### 3.8 Others

Apart from the abovementioned cancer types, miR‐204‐5p also plays an inhibitory role in other human tumors, including cervical cancer,^92^ osteosarcoma,^93^ pancreatic cancer,^94^, ^95^ and renal cell carcinoma.^96^, ^97^ In cervical cancer, miR‐204 inhibits tumor progression via regulating cell proliferation, apoptosis and autophagy.^35^, ^98^ In renal cell carcinoma, SNHG4^96^ and HOTAIR^97^ antagonized miR‐204‐5p to accelerate tumor proliferation and invasion. MiR‐204‐5p also inhibits tumorigenesis and progression in pancreatic cancer. For example, miR‐204 suppresses proliferation, migration, and invasion in pancreatic cancer by targeting MCL‐1^94^ and RACGAP1^95^; lncRNA ZEB2‐AS1 accelerates tumor growth and invasion by regulating the miR‐204/HMGB1 axis.^27^

## 4 MIR‐204‐5P REGULATES TUMORIGENESIS AND PROGRESSION BY TARGETING MULTIPLE KEY SIGNALING PATHWAYS

**FIGURE 1:**
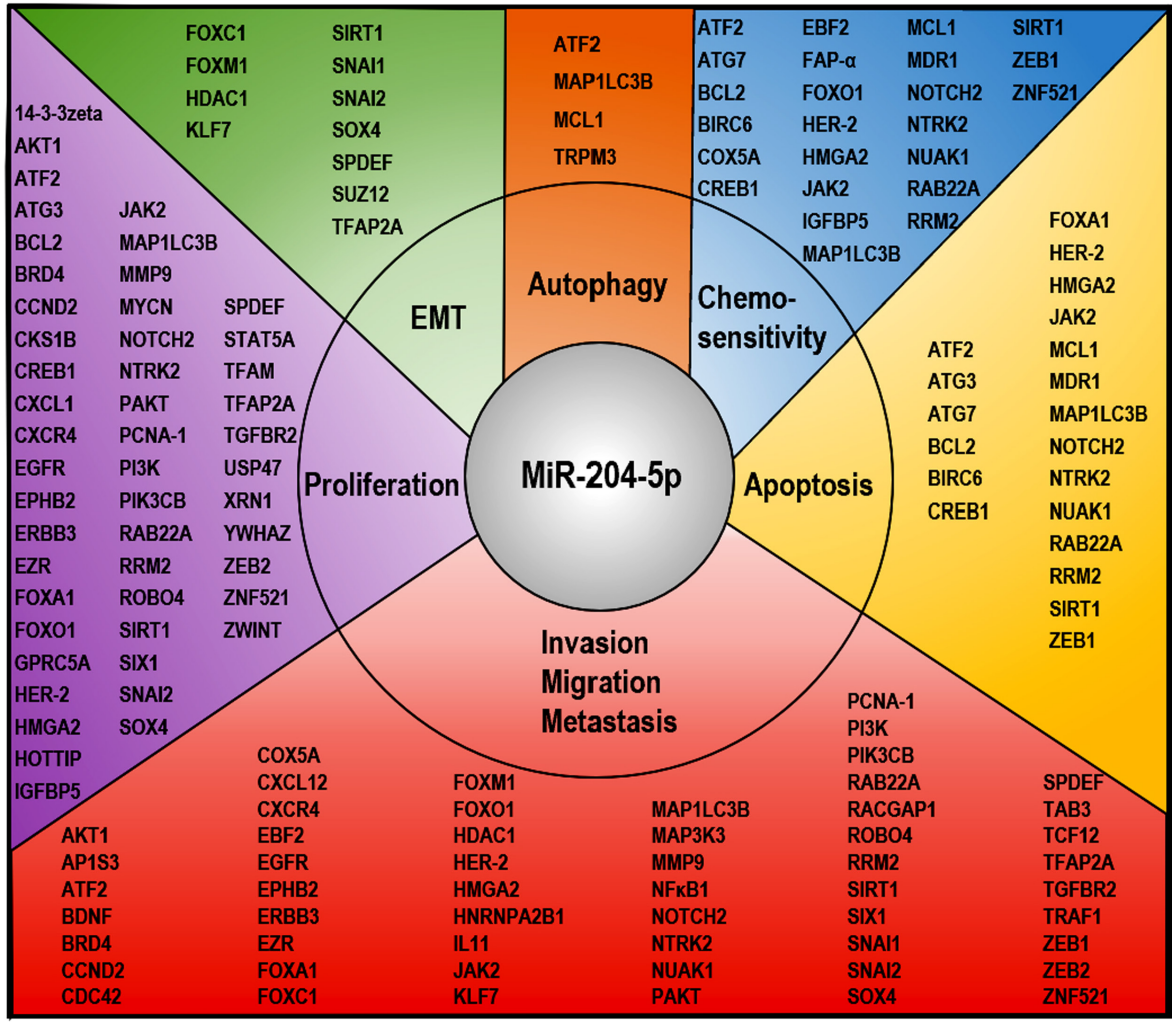
MiR‐204‐5p regulates tumorigenesis and progression via various mRNA targets in human cancers. This figure summarizes miR‐204‐5p targets shown in Table 1 of this paper.

Sustaining proliferation signaling, activating invasion and metastasis, and resisting cell death are hallmarks of cancer cells. As mentioned above, as an essential tumor suppressor, miR‐204‐5p regulates cell proliferation, metastasis, invasion, autophagy, apoptosis, and chemoresistance by inhibiting dozens of target genes, demonstrating the extensive and universal functions of miR‐204‐5p (Tables 1 and 2 and Figure 1). For example, BCL2 has been identified as the target gene of miR‐204‐5p in different types of tumors, including CRC,^99^ GC,^100^ glioma,^86^ neuroblastoma,^101^ intrahepatic cholangiocarcinoma,^81^ PCa^49^ and HCC.^74^ Next comes SOX4, which has been identified as a target of miR‐204‐5p in BC,^39^ CRC,^47^ GC,^61^ glioma,^21^ and oral squamous cell carcinoma.^102^ When it comes to the number of miR‐204‐5p targets identified, to the best of our knowledge, at least 16 genes have been reported in GC. These studies indicate that miR‐204‐5p, as the core of a regulatory network, plays a tumor‐inhibitory role by regulating a large group of target genes in pan‐cancer.

**TABLE 1:** Coding RNA target genes of miR‐204‐5p

Target genes	Cancer types	Function	Reference
14–3‐3zeta	OS	‐proliferation	^112^
AKT1	BC, ESCA	‐proliferation and metastasis	37, 103
ANGPT1	BC	‐angiogenesis	^38^
AP1S3	BC	‐migration, and invasion	^42^
ATF2	PCa, Glioma, CC, NSCLC	‐proliferation, metastasis, autophagy, migration, and apoptosis	53, 85, 98, 113
ATG3	NSCLC	‐proliferation and apoptosis	^114^
ATG7	OC	‐apoptosis	^115^
BCL2	PCa, HCC, ICC, CRC, GC, NB, Melanoma, RB, BC	‐chemosensitivity, apoptosis, and proliferation	49, 74, 81, 99, 100, 101
BDNF	BC	‐migration and invasion	^43^
BIRC6	AML	‐apoptosis	^116^
BRD4	TSCC	‐proliferation, migration, and invasion	^117^
CCND2	RB	‐proliferation and invasion	^118^
CDC42	NPC	‐invasion and metastasis	^31^
CKS1B	GC	‐proliferation	^59^
COX5A	BC	‐invasion, metastasis, and chemoresistance	^40^
CREB1	CRC	‐proliferation and apoptosis	^28^
CXCL1	GC	‐proliferation	^59^
CXCL12	GC	‐metastasis	^60^
CXCR4	GC, OSCC, NPC	‐metastasis and proliferation	60, 119, 120
E2F1	HCC	‐cell cycle	^80^
EBF2	OS	‐apoptosis and migration	^93^
EGFR	GC	‐migration and proliferation	^121^
EPHB2	Glioma, CC	‐migration and stemness	21, 122
ERBB3	GC	‐invasion, proliferation, and metastasis	^57^
EZR	Glioma, GC	‐proliferation, migration, and invasion	87, 123
FAP	Glioma	‐chemoresistance	^88^
FOXA1	BC	‐proliferation, migration, invasion, and apoptosis	^124^
FOXC1	EEC, LSCC	‐metastasis, migration, invasion, and EMT	125, 126
FOXM1	ESCA	‐invasion and EMT	^127^
FOXO1	BC	‐different alterations of cellular activity	^128^
GPRC5A	GC	‐proliferation	^59^
HDAC1	HNSCC	‐EMT	^129^
HER‐2	GC	‐proliferation, migration invasion, and apoptosis	^130^
HMGA2	CRC, OSCC, THCA, ESCA	‐chemosensitivity, proliferation, and metastasis	131, 132, 133, 134
HNRNPA2B1	BC	‐migration and invasion	^17^
HOTTIP	HCC	‐proliferation	^78^
HOXA10	AML	‐regulation	^135^
IGFBP5	PTC	‐proliferation and apoptosis	^136^
IL11	BC, ESCA	‐metastasis and invasion	44, 137
JAK2	NSCLC, LC, BC, HNSCC	‐proliferation, invasion, and migration	68, 69, 138, 139
KHDRBS1	BC	‐self‐renewal	^140^
KLF7	NSCLC	‐migration, invasion, and EMT	^141^
MAP1LC3B	OC, ccRCC	‐proliferation, chemosensitivity, and apoptosis	^115^
MAP3K3	PCa	‐invasion, migration, and metastasis	^14^
MCL1	PC	‐apoptosis and autophagy	^94^
MDR1	OC	‐apoptosis	^115^
MEIS1	AML, Nephroblastoma	‐tumorigenesis	135, 142
MET	OC	‐cell infiltration	^143^
MMP9	RB	‐proliferation and invasion	^118^
			
MYCN	NB	‐proliferation and tumorigenesis	^144^
			
NFκB1	PCa	‐invasion, migration, and metastasis	^14^
NOTCH2	GBC	‐proliferation, invasion, and apoptosis	^106^
NTRK2	ESCA, NB	‐proliferation, invasion, and chemosensitivity	32, 101
NUAK1	NSCLC, HCC	‐metastasis and chemosensitivity	70, 75
PAKT	BC	‐proliferation and metastasis	^37^
PCNA‐1	LC	‐proliferation, migration, and invasion	^145^
PHOX2B	NB	‐regulation	^146^
PI3K	BC, ESCA	‐proliferation and metastasis	37, 103
PIK3CB	BC	‐metastasis, proliferation, and migration	^16^
PTEN	BC	‐regulation	^37^
PTPN11	cSCC	‐migration	^20^
RAB22A	GC, Glioma, RCC	‐proliferation, invasion, and chemosensitivity	58, 84, 147
RACGAP1	PDAC	‐migration and invasion	^95^
ROBO4	Bladder Cancer	‐growth and metastasis	^148^
RRM2	BC	‐proliferation, metastasis, and apoptosis	^25^
RUNX2	PCa	‐regulation	^149^
SIRT1	PCa, GC, HCC, RB	‐proliferation, invasion, apoptosis, EMT, anoikis resistance, and chemosensitivity	52, 62, 76, 150
SIX1	NSCLC, HCC, BC	‐proliferation and invasion	71, 77, 151
SNAI1	GC	‐EMT, metastasis, and invasion	^152^
SNAI2	ICC, OSCC, HNSCC	‐metastasis, EMT, stemness, and self‐renewal	82, 102, 129
SOX4	Glioma, BC, CRC, GC, OSCC, LC, T‐ALL, RCC	‐stemness, proliferation, migration, invasion, metastasis, and EMT	21, 39, 47, 61, 102, 153, 154, 155
SPDEF	PCa, BC	‐migration, invasion, metastasis, and EMT	11, 12
STAT3	HNSCC	‐regulation	^121^
STAT5A	B‐cell lymphoma	‐proliferation	^156^
SUZ12	HNSCC	‐EMT	^121^
TAB3	PCa	‐invasion, migration, and metastasis	^14^
TCF12	CC	‐migration and invasion	^92^
TFAM	CRC	‐proliferation	^157^
TFAP2A	CC	‐proliferation, migration, invasion, and EMT	^35^
TGFBR2	BC	‐proliferation, migration, and angiogenesis	^38^
THBS1	OC	‐angiogenesis	^13^
TRAF1	PCa	‐invasion, migration, and metastasis	^14^
TRPM3	ccRCC	‐autophagy	^158^
USP47	OC, GC	‐proliferation	18, 58
XRN1	PCa	‐proliferation	^51^
YWHAZ	ESCA	‐growth	^103^
ZEB1	PCa, PC	‐migration, invasion, chemosensitivity, and apoptosis	159, 160
ZEB2	BC, HCC	‐growth, migration, and invasion	45, 161
ZNF521	GC	‐apoptosis, proliferation, migration, and invasion	^56^
ZWINT	BC	‐proliferation	^162^

**TABLE 2:** Non‐coding RNA targets of miR‐204‐5p

	Target genes	Cancer types	Function	Reference
LncRNA	ARNILA	BC	‐EMT, invasion, and metastasis	^39^
	ATXN8OS	BC	‐proliferation, viability, and invasion	^163^
	BANCR	Melanoma	‐growth and invasion	^164^
	BCYRN1	GC	‐proliferation, migration, and invasion	^63^
	BRM	OC	‐proliferation, migration, and invasion	^165^
	DGUOK‐AS1	BC	‐migration, angiogenesis, and metastasis	^46^
	DLX6‐AS1	GC	‐proliferation, migration, invasion, and EMT	^64^
	DNM3OS	Oral cancer	‐viability and migration	^166^
	DSCAM‐AS1	BC, CRC	‐proliferation and apoptosis	25, 47
	HOTAIR	RCC, ESCA, CCA,	‐invasion, migration, apoptosis, autophagy, and proliferation	97, 190, 191
	HOTTIP	HCC	‐viability and proliferation	^78^
	HOXD‐AS1	Glioma	‐proliferation, migration, invasion, and cisplatin sensitivity	^91^
	KCNQ1OT1	NSCLC, MSSCC	‐proliferation, migration, and invasion	114, 167
	MALAT1	BC, LC, HCCA, TC, HCC, GC,	‐migration, invasion, EMT, autophagy, and proliferation	45, 73, 83, 168, 169, 170
	MIR100HG	LSCC	‐proliferation, migration, and invasion	^171^
	NEAT1	PCa, NSCLC, HCC, RB, NPC,	‐proliferation, migration, invasion, apoptosis, EMT, radioresistance, sorafenib resistance, autophagy, and docetaxel resistance	55, 72, 79, 172, 173
	OIP5‐AS1	LSCC	‐proliferation, migration, invasion, and EMT	^174^
	PBB12	OS	‐proliferation and invasion	^175^
	PCAT6	CRC	‐chemoresistance	^26^
	PlncRNA‐1	CRC	‐proliferation and metastasis	^48^
	ROR	ESCA	‐apoptosis	^176^
	SNHG1	ESCA	‐migration, invasion, and apoptosis	^177^
	SNHG4	GC, RCC	‐proliferation, metastasis, migration, invasion, and EMT	65, 96, 178
	SNHG6	HCC	‐cell cycle and proliferation	^80^
LncRNA	UCA1	CRC, PCa, Glioma, CC, AML, ESCA, PTC	‐proliferation, invasion, docetaxel sensitivity, apoptosis, migration, and EMT	28, 52, 53, 54, 90, 179, 180, 181, 182, 183
	XIST	Glioma, RB	‐proliferation, autophagy, vincristine sensitivity, migration, and invasion and apoptosis	86, 184
	ZEB2‐AS1	PC	‐growth, cell cycle, and invasion	^27^
	LINC00518	Melanoma	‐metastasis	^185^
	LINC01234	GC	‐apoptosis and growth	^66^
CircRNA	Circ0021205	CCA	‐proliferation, migration, and invasion	^30^
	CircPVT1	BC	‐invasion and EMT	^29^
	CircSLAMF6	GC	‐glycolysis, migration, and invasion	^67^
	Circ0001971	OSCC	‐proliferation, migration, invasion, apoptosis, and chemosensitivity	^186^
	CircMTO1	RCC	‐proliferation, migration, invasion, and apoptosis	^187^
	CircNOP10	GC	‐proliferation, migration, and EMT	^188^
	Circ‐E2F3	RB	‐proliferation, migration, invasion, and apoptosis	^189^

**FIGURE 2:**
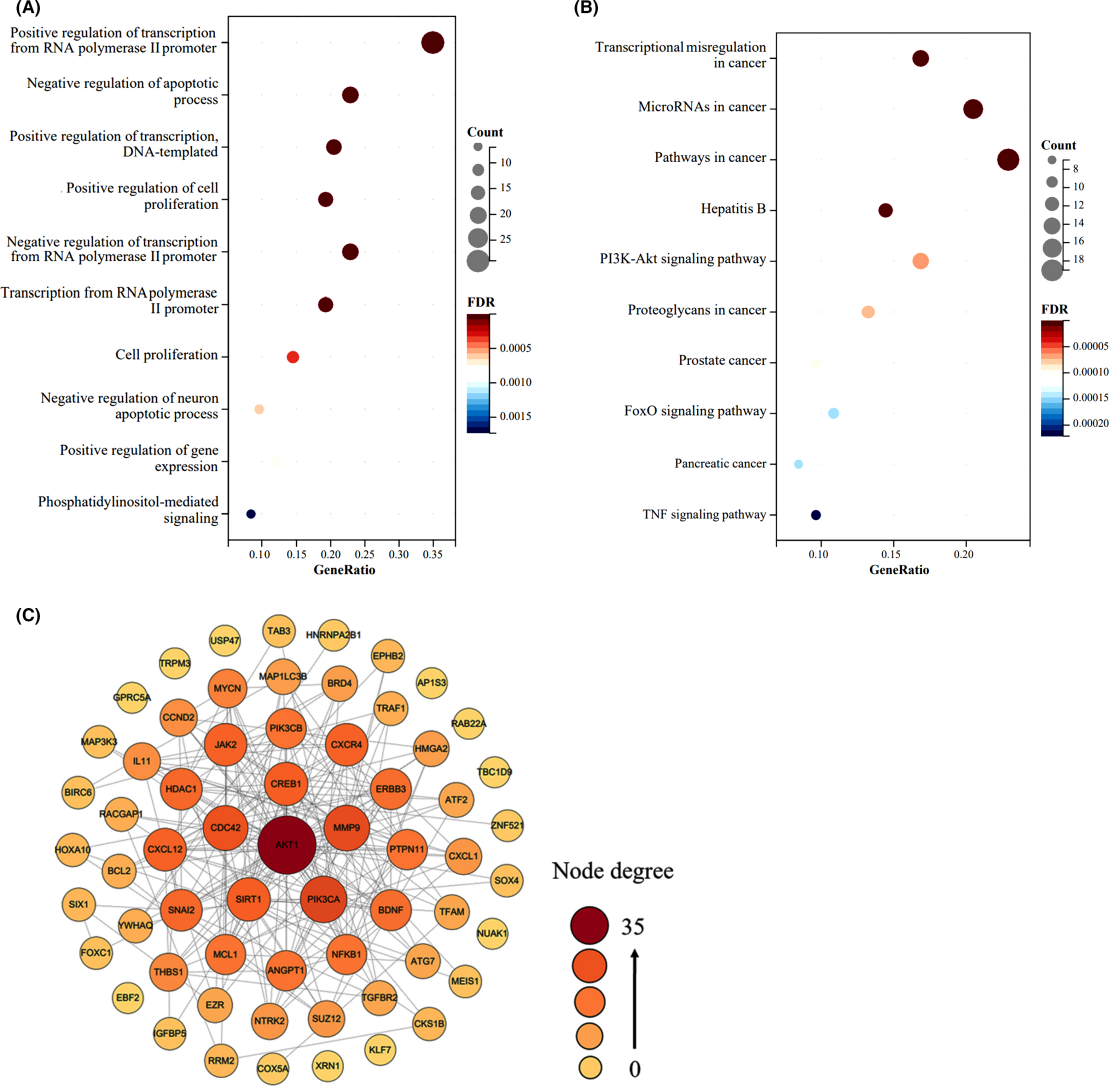
MiR‐204‐5p regulates multiple pathways in human cancers. (A and B) Gene ontology (GO) Analyses of miR‐204‐5p targets using the online tool Sangerbox (http://vip.sangerbox.com/). (B) Kyoto Encyclopedia of Genes and Genomes (KEGG) enrichment analyses of miR‐204‐5p targets using Sangerbox. (C) Protein–protein interaction (PPI) network was constructed using the mRNA targets of miR‐204‐5p. The web tool (https://cn.string‐db.org/) and the software Cytoscape 3.8.2 were applied to construct the PPI network.

To better understand the role of miR‐204‐5p in human cancers, we applied the online tool Sangerbox (http://vip.sangerbox.com/) to perform Gene Ontology (GO) and Kyoto Encyclopedia of Genes and Genomes (KEGG) pathway enrichment analyses using on all identified mRNA targets of miR‐204‐5p (Table 1). GO enrichment results showed these target genes mainly participated in the transcription regulation, cell proliferation, and apoptosis (Figure 2A). KEGG pathway analyses indicated that they were mainly enriched in Transcriptional Dysregulation in Cancer, PI3K/AKT and other cancer‐related pathways (Figure 2B). In addition, we constructed a PPI network by using the STRING database (https://cn.string‐db.org/) and Cytoscape 3.8.2 software. In the network, there were 84 nodes and 554 edges, with an average node degree of 13.2 and a local clustering coefficient of 0.539 (Figure 2C). In the PPI network, the core targets, including AKT1, CREB1, CDC42, SIRT1, PIK3CA, and MMP9, are key cancer‐related genes, which further indicate the important role of miR‐204‐5p in human cancers (Figure 2C).

Apoptosis is one of the most common phenotypes regulated by miR‐204‐5p and its target genes. BCL2 is a key apoptosis regulator and is frequently upregulated in cancer cells. As summarized above, BCL2, the most common target of miR‐204‐5p, was reported to be regulated by miR‐204‐5p in seven different types of tumors. In these cancer types, miR‐204‐5p enhances apoptosis and reverses chemoresistance by inhibiting BCL2 expression.

The other representative pathway enriched from miR‐204‐5p targets is PI3K/AKT which plays a critical role in cell proliferation, survival, migration, differentiation, angiogenesis, and metabolism. Due to its key role in these critical cellular processes, dysregulation of PI3K/AKT pathway is closely associated with many human diseases, especially cancers. Some members of this pathway, including the two most important factors of PI3K/AKT pathway AKT1 and PI3K, are reported to be direct targets of miR‐204‐5p in multiple cancer types. Aberrant activation of PI3K/AKT pathway due to miR‐204‐5p induces apoptosis inhibition, chemoresistance, migration, invasion, and angiogenesis in cancer cells.^37^, ^103^

## 5 POTENTIAL CLINICAL APPLICATIONS OF MIR‐204‐5P FOR CANCERS

Decreased expression of miR‐204‐5p in cancer tissues often predicts poor therapeutic effects and prognosis, strongly suggesting it as a cancer biomarker. MiR‐204‐5p has been reported as a prognostic factor in 20 types of malignancies in a TCGA‐based study.^104^ Most studies indicate the protective role of overexpressed miR‐204‐5p. For example, in two independent melanoma cohorts, miR‐204 expression was associated with a better prognosis.^105^ In addition, a low level of miR‐204 is related to poor prognosis in CRC,^22^ GC,^100^ BC^40^, and some other cancers.^31^, ^106^, ^107^

In addition, accumulated data have confirmed the extensive anti‐cancer functions of miR‐204‐5p. Consequently, restoring the expression and tumor‐suppressive effects of miR‐204‐5p may be a promising strategy for cancer therapy. For example, drug resistance is a major obstruction to successful cancer treatment, and the strong chemotherapeutic sensitization effect of miR‐204‐5p highlight a new strategy to reverse drug resistance and improve chemotherapeutic efficacy. Many studies have confirmed that ectopic miR‐204‐5p expression increases the response of cancer cells to chemotherapeutic agents, including 5‐FU and oxaliplatin.

Due to the small molecular weight and high stability, miRNAs have been suggested as promising therapeutic molecules. Even though miRNAs have a great potential for cancer treatment, we should pay attention to it possible adverse reactions. Recently, a miR‐34a‐based clinical trial for cancer treatment was stopped by FDA on account of immune‐mediated toxicities, reflecting the importance of targeting the delivery system.^107^ An ideal delivery system for miRNA‐based therapy should meet at least the following five requirements which we summarize as “three high and two low” characteristics: high affinity, high specificity, high stability, low cost, and low side effects.

Recently, different strategies, including aptamers, nanoparticles, and cell‐penetrating peptides, have been tried to deliver miRNAs. Zheng et al. developed PEGylated polymer nanoparticle for delivering miR‐204‐5p, which showed an obvious tumor‐inhibitory effect in a CRC xenograft model.^109^ Fattore et al. confirmed the anti‐tumor efficiency of encapsulated miR‐204‐5p by lipid nanoparticles in melanoma.^110^ Compared with these artificial materials, exosome appears to be a promising drug delivery carrier for its unique features, including low toxicity, immune compatibility, nanoscale size, and circulation stability in vivo. We showed that exosome‐encapsulated miR‐204‐5p significantly inhibits CRC growth and chemoresistance without obvious side effects.^191^ In addition, due to the numerous miR‐205‐5p targets identified, when designing clinical trials, we should keep in mind that miR‐204‐5p may regulate different phenotypes in different cancer types by regulating different targets.

## 6 PERSPECTIVES

Due to their extensive regulatory functions for gene expression, miRNAs have been extensively studied in human diseases, especially cancers.^14^, ^22^, ^40^ Several clinical trials have been performed to evaluate the value of miRNAs as cancer biomarkers or therapeutic targets.^40^, ^109^, ^110^ As a pivotal tumor suppressor, miR‐204‐5p shows the latent clinical value for predicting cancer prognosis and therapeutic efficacy. However, multiple centers' clinical trials should be performed to evaluate the clinical importance of miR‐204‐5p before it is considered as a cancer biomarker.

Targets regulated by miR‐204‐5p formed a large network to affect cancer cell proliferation, metastasis, angiogenesis, apoptosis, and chemosensitivity, suggesting miR‐204‐5p as a candidate therapeutic molecule for cancers. As abovementioned, several groups have shown the broad perspective of miR‐204‐5p for cancer therapy.^109^, ^110^, ^111^ However, to the best of our knowledge, there is still no clinical trial performed to evaluate the therapeutic efficacy of miR‐204‐5p in cancers. We hope that this review can promote further research on miR‐204‐5p to understand its biological role in human cancers and provide a theoretical foundation for the clinical application of miR‐204‐5p.

## AUTHOR CONTRIBUTION

Zhaohui Huang, Fan Yang, and Zehua Bian designed and wrote the manuscript. Peiwen Xu collected and analyzed references. Fan Yang and Shengbai Sun participated in bioinformatic analyses.

## FUNDING INFORMATION

This study was partially supported by grants from the Social Development Project of Jiangsu Province (BE2019632), the Six Talent Peaks Projects of Jiangsu Province (WSW‐WSW‐196), Wuxi Taihu Lake Talent Plan, and Wuxi Medical Key Discipline (ZDXK2021002).

## CONFLICT OF INTEREST

No potential conflict of interest was reported by the author(s).

## Data Availability

None.
